# Supplementary material for: Bringing weak transitions to light
Source: Nat Commun. 2025 Jun 20;16:5322. doi: 10.1038/s41467-025-60701-9 (PMC12181367; doi:10.1038/s41467-025-60701-9)
Supplement: Supplementary file 1 — Supplementary Information [file 41467_2025_60701_MOESM1_ESM.pdf]

Supplementary information for
Bringing Weak Transitions to Light

Yu He,[1*] Xiao-Min Tong,[2] Shuyuan Hu,[1] Gergana D. Borisova,[1] Hao Liang,[3]
Maximilian Hartmann,[1] Veit Stooß,[1] Chunhai Lyu,[1] Zoltán Harman,[1]
Christoph H. Keitel,[1] Kenneth J. Schafer,[4] Mette B. Gaarde,[4]
Christian Ott,[1*] and Thomas Pfeifer[1*]

[1]Max-Planck-Institut für Kernphysik, Saupfercheckweg 1, 69117 Heidelberg, Germany

[2]Center for Computational Sciences, University of Tsukuba, 1-1-1 Tennodai, Tsukuba, Ibaraki 305-8573, Japan

[3]Max-Planck-Institut für Physik komplexer Systeme, Nöthnitzer Straße 38, 01187 Dresden, Germany

[4]Department of Physics and Astronomy, Louisiana State University, Baton Rouge, Louisiana 70803, USA

[*]E-mail: yuhe@mpi-hd.mpg.de; christian.ott@mpi-hd.mpg.de; thomas.pfeifer@mpi-hd.mpg.de

Supplementary Table 1: **Absolute values of the related transition matrix elements (in atomic units).**

Transitions	This work	Wang *et al.*[1]
$1s^2\ (^1S^e) - 2s2p\ (^1P^o)$	0.0365	0.026
$1s^2\ (^1S^e) - sp_{2,3+}\ (^1P^o)$	0.0145	0.017
$1s^2\ (^1S^e) - 2p3d\ (^1P^o)$	0.0008	
$1s^2\ (^1S^e) - sp_{2,4-}\ (^1P^o)$	0.0015	
$1s^2\ (^1S^e) - sp_{2,4+}\ (^1P^o)$	0.0091	
$2p^2\ (^1S^e) - 2s2p\ (^1P^o)$	2.11	1.99
$2p^2\ (^1S^e) - 2p3d\ (^1P^o)$	1.74	
$2p^2\ (^1S^e) - sp_{2,4-}\ (^1P^o)$	1.13	

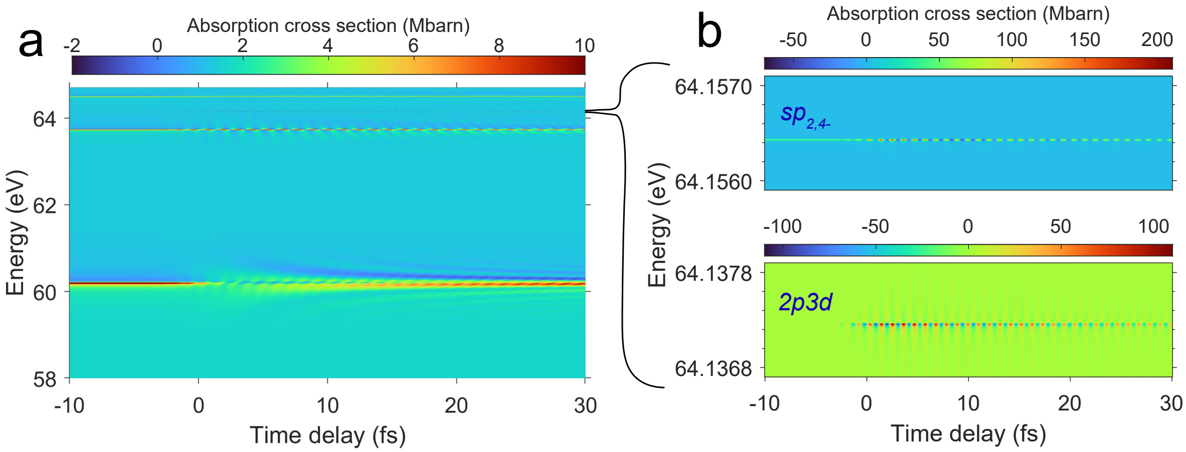

Supplementary Figure 1: **Theoretical results. a** Calculated time-resolved generalized absorption cross section and **b** the close-up around the $2p3d$ and $sp_{2,4-}$ states within an energy range of 1.2 meV. The generalized absorption cross section of both $2p3d$ and $sp_{2,4-}$ states gets substantially enhanced when the XUV pulse overlaps or precedes the VIS pulse.

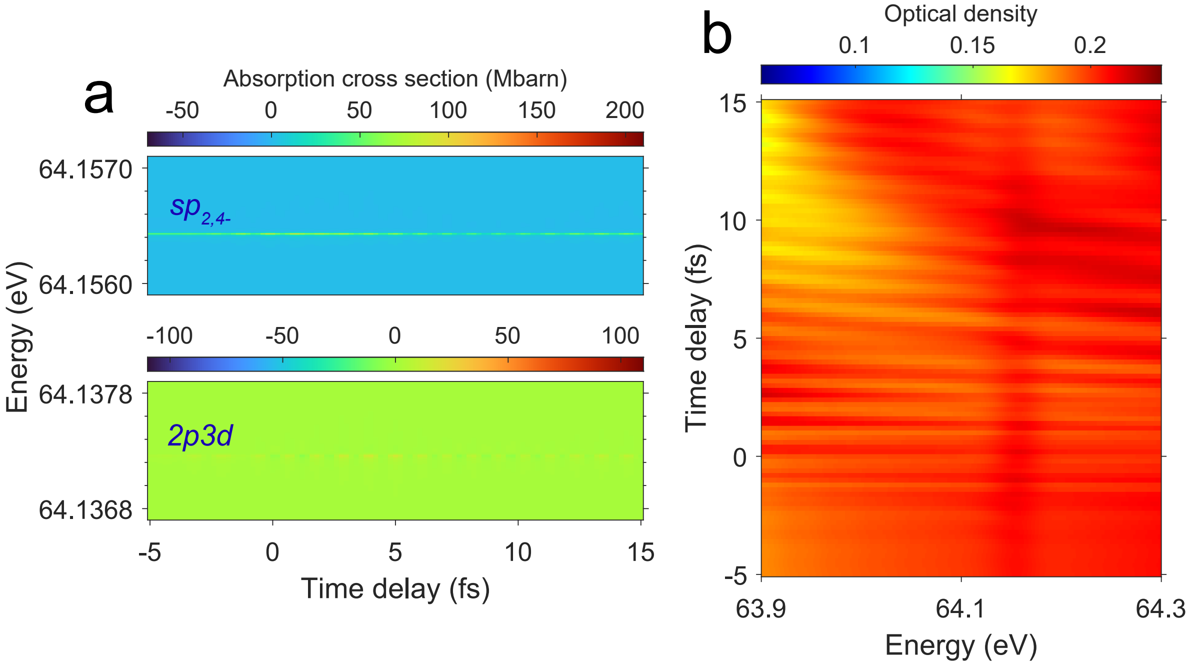

Supplementary Figure 2: **Identification of the dominant pathway for the enhanced spectral visibility.** **a** Time-resolved generalized absorption cross section obtained by artificial removal of the $2p^2$ ($^1S^e$) state in the numerical simulation. **b** The resulting transient absorption spectrum. For a direct comparison, the color scales for the cross section and the optical density are kept the same as those in the full numerical results in Supplementary Fig. 1b and Fig. 3b in the main manuscript, respectively. Without the intermediate $2p^2$ ($^1S^e$) state, the two-VIS-photon coupling between $2s2p$ and $2p3d/sp_{2,4-}$ ($^1P^o$) states is highly suppressed and no obvious resonant feature shows up in the absorption spectrum.

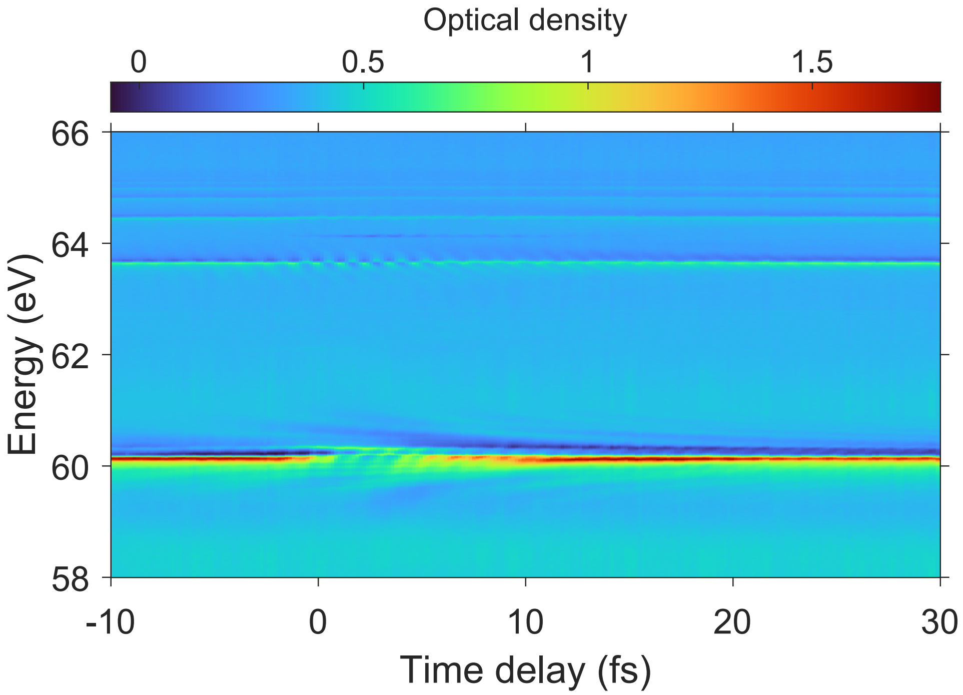

Supplementary Figure 3: **Measured transient absorption spectrum in a large energy range.** Contrary to the spectral evolution of $2p3d/sp_{2,4-}$ states shown in Fig. 3 in the main manuscript, the spectral amplitude of $2s2p$ state drops significantly in the pulse-overlap region, and then gradually rises with increasing time delay. This is related to the loss of amplitude of $2s2p$ state by the action of the coupling VIS pulse when it arrives at the helium target after the XUV pulse.

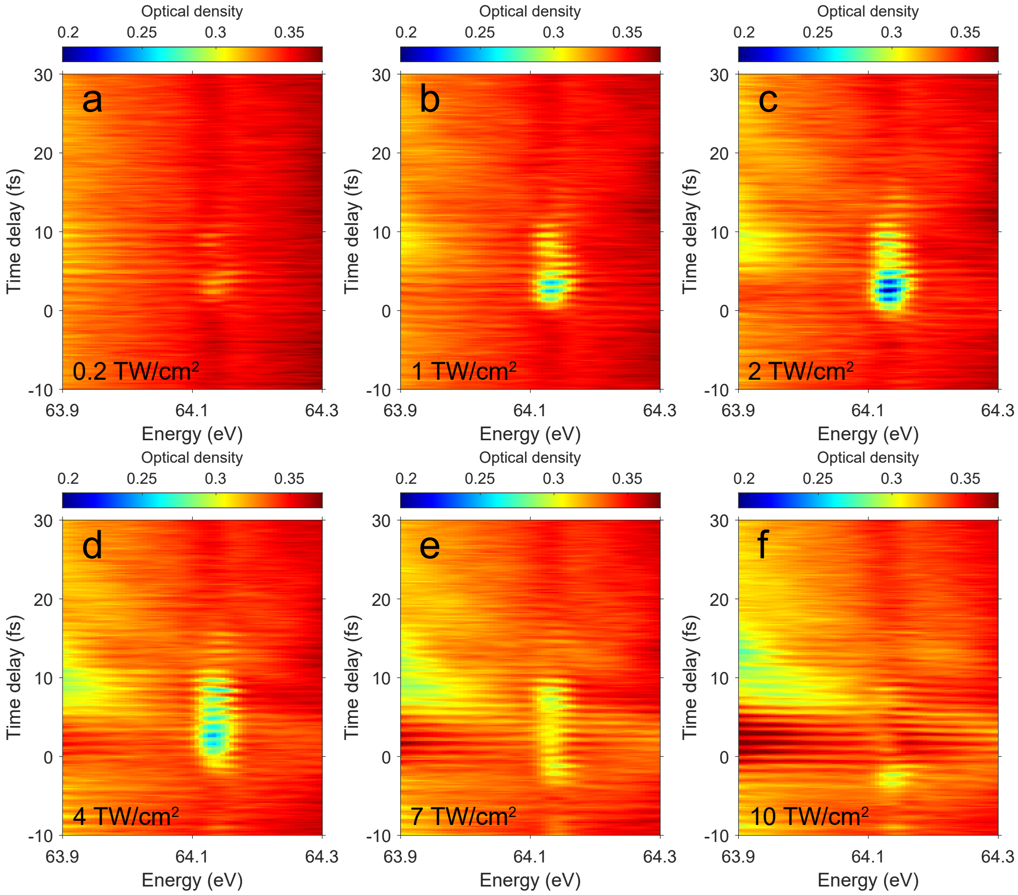

Supplementary Figure 4: **Effects of the VIS intensity on the spectral enhancement of $2p3d/sp_{2,4-}$ states.** The spectral visibility first increases with increasing VIS intensity (panels **a**–**c**) since more quantum-state amplitude can be transferred to these two states, which subsequently decreases (panels **d**–**f**) due to the ionization loss of the $2s2p$ and $2p3d/sp_{2,4-}$ states at high VIS intensities. Pronounced spectral enhancement is shown in an energy range of 1–7 $\mathrm{TW/cm^2}$.

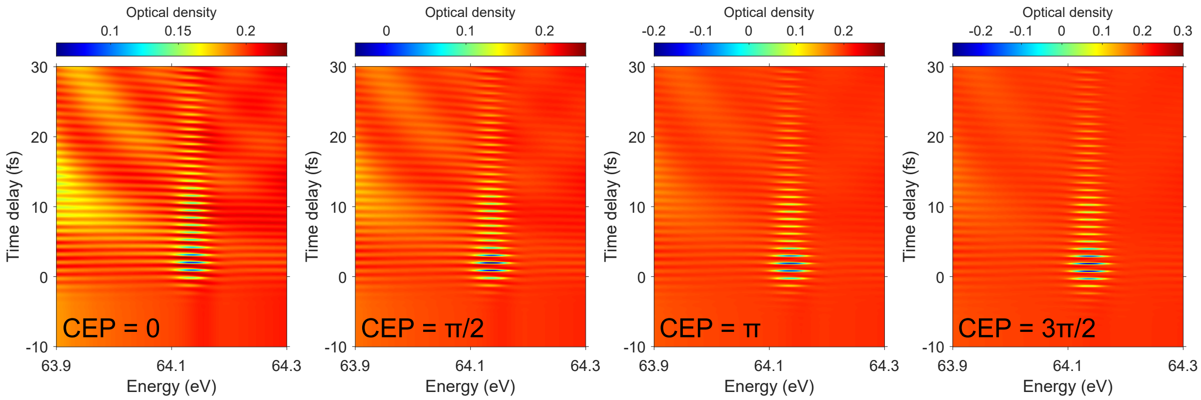

Supplementary Figure 5: **Effects of the carrier-envelope phase (CEP) of the VIS pulse.** Differences in resonant OD values are observed by varying the VIS light CEP, while the general spectral evolution with respect to time delay is pretty robust and the oscillations are still in phase.

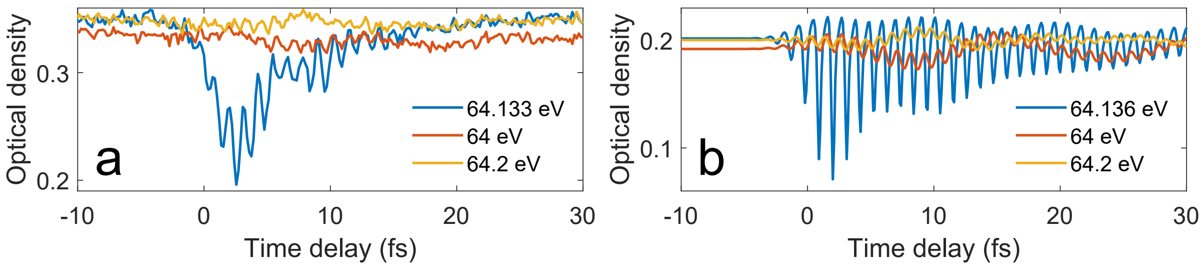

Supplementary Figure 6: **OD lineouts as a function of time delay at three different energy positions.** Except for a lower contrast of the oscillation observed in the experiment results in panel **a**, which is explained by experimental time-delay jitter and spatial beam inhomogeneity, the observed features are well captured by the theory calculations in panel **b**.

Supplementary References

1. Wang, Y.-S., Kar, S. & Ho, Y. K. Dipole transition elements and oscillator strengths for the doubly excited states in the helium atom. *Chem. Phys. Lett.* **774,** 138640 (2021).
